# A new paradox for well-being subjectivism

**DOI:** 10.1093/analys/anad040

**Published:** 2023-08-25

**Authors:** Ben Davies

**Affiliations:** Uehiro Centre for Practical Ethics, University of Oxford UK

## Abstract

Subjectivists think that our well-being is grounded in our subjective attitudes. Many such views are vulnerable to variations on the ‘paradox of desire’, where theories cannot make determinate judgements about the well-being of agents who take a positive valuing attitude towards their life going badly. However, this paradox does not affect all subjectivist theories; theories grounded on agents’ prudential values can avoid it.

This paper suggests a new paradox for subjectivist theories which has a wider scope, and includes such prudential judgement theories. I outline the new paradox and show how two plausible idealisztions (coherence and consideration) will not help. Subjectivists about well-being must either add an additional idealization that can solve the paradox of judgement or explain why such paradoxes do not constitute serious objections to a theory of well-being.

## 1. Introduction

Subjectivists think a subject *S*’s well-being is determined by *S*’s subjective attitudes. A common candidate is desire (Barrett 2022, Heathwood 2006, 2022, Lin 2016, Murphy 1999). The simplest desire view is that *S*’s well-being depends on *S* getting what she desires.

Many subjectivist views face a paradox: the ‘paradox of desire’. However, views which ground well-being in what *S prudentially values* avoid it (e.g. Dorsey 2012, 2021, Tiberius 2018). Thus, avoidance of paradox is one point in favour of subjectivism based on judgements of prudential value over rival subjectivist views: call such views ‘judgement subjectivism’ or JS.

This paper outlines a new paradox for subjectivism. Like the paradox of desire, it applies to a wide range of subjectivist views; unlike the paradox of desire, this includes subjectivist views grounded in what agents judge prudentially valuable. After outlining the paradox of desire and showing how JS avoids it, I outline the new paradox which affects JS along with a range of other subjectivist views. I show that two idealizations adopted by leading JS theorist Dale Dorsey, and which might plausibly be adopted by a range of subjectivist views, will not help to avoid this new paradox.

The paradox of desire is outlined by Bradley 2007 (see also 2009: 30–32, Feldman 2004: 17, Heathwood 2005). DS stands for ‘desire-satisfactionism’:

Suppose DS is true, and suppose Epimenides has just two desires. His first desire, D_a_, is a desire of intensity +5 for an apple. He does not get the apple, so his life includes a desire frustration of value –5. His second desire, D_b_, is a desire of intensity +10 that his life goes badly for him. Is D_b_ satisfied? If it is, then Epimenides’ life contains a desire-satisfaction of value +10, in which case his life has an overall value of +5 (it goes well for him), in which case D_b_ is not satisfied after all. If D_b_ is not satisfied, then his life contains a desire frustration of value –10, in which case his life has an overall value of –15 (it goes badly for him), in which case D_b_ is satisfied. Thus if DS is true, D_b_ is satisfied if and only if it is not satisfied, and Epimenides’ life goes well if and only if it does not go well. (Bradley 2007: 46)

Desire theorists cannot avoid the paradox; nor can many other potential attitude-based theories of well-being. But so long as the prudential judgements grounding well-being are required to be coherent, JS can avoid an equivalent challenge (Dorsey 2012: 422–24).

To generate a judgement-translated equivalent to the paradox of desire, I swap references to desires for references to Epimenides’ coherent judgements about what is intrinsically good for him. I translate ‘desires of intensity *N*’ to ‘judgements of value *N*’.

The translated scenario is:

Suppose JS is true. Epimenides forms just two considered judgements about what is intrinsically good for him. His first judgement, J_a_, is a judgement of value +5 that eating an apple would be intrinsically good for him. He does not get the apple, so his life includes a judgement frustration of value –5. His second judgement, J_b_, is a judgement of value +10 that it would be intrinsically good for him if his life goes badly for him.

Whereas this scenario led to a paradox concerning desires, JS makes this scenario non-paradoxical because it is incoherent to judge that your life going badly for you is intrinsically good for you. It is not coherent to judge that something being intrinsically bad for you is intrinsically good for you.^1^ The paradox does not arise. Proponents of JS can offer a determinate judgement about how well Epimenides’ life went for him: it was net negative, since his sole relevant prudential value judgement was frustrated.

## 2. The new paradox

JS can avoid the judgement-translated paradox of desire. However, a related paradox is not far away. Since JS is the subjective theory which most obviously escapes the paradox of desire, I formulate this new paradox in terms of intrinsic prudential judgements. I also show that the new paradox affects a range of subjective attitudes, and thus represents a significant problem for subjectivists.

Consider:

Pythagoras forms various considered judgements about what is intrinsically good for him. Over the course of his life, exactly nine are satisfied; the rest are frustrated. Given their respective values, Pythagoras’s well-being just before his death is neutral. His final considered judgement, which he has held for most of his life, is that it would be good for him if fewer than ten of his judgements about what is good for him are satisfied. Call this final judgement, *Ten*.^2^

Given the facts about his previous judgements, *Ten* may initially seem to be satisfied: Pythagoras has had nine relevant judgements satisfied, fewer than ten. But then *Ten* itself would be satisfied, taking his total to ten, and so on.

The paradox occurs at two levels. First, it is unclear whether *Ten* has been satisfied. Second, if Pythagoras’s lifetime welfare is neutral apart from *Ten*, JS cannot say whether Pythagoras’s lifetime well-being is positive, negative or neutral. Pythagoras’s attitude is odd, but not unrealistic. Many people ascribe value to particular numbers.^3^ Perhaps Pythagoras thinks that there is something bad about the number ten, and it is best to avoid it. The judgement is also stable across his life: he avoids going over ten wherever he can (he keeps his stamp collection meagre, refuses to rent homes numbered above ten etc.). Importantly, Pythagoras need not be *overly* unrealistic to make the new paradox work. He may be just like any other individual, forming a range of ‘ordinary’ subjective attitudes alongside *Ten*: the paradox arises if those other attitudes balance out to make his well-being neutral. So, we cannot dismiss him as irrelevantly unrealistic.

I will shortly consider whether certain idealizations can rescue JS from paradox. However, I first show that the paradox applies more widely.

Consider desire again. If Pythagoras has had nine desires satisfied, and forms a tenth desire that fewer than ten of his desires are satisfied, an analogous problem arises. So too for the claim that Pythagoras ‘prefers’ *Ten* to be satisfied over its not being satisfied (Barrett 2019), and for several of the ‘positive attitudes’ mentioned by Heathwood (2014: 202), including ‘caring about it … having it as a goal, being fond of it, being for it’. Insofar as these attitudes can be taken at a higher order (e.g. having goals about your goals), the paradox can arise. Finally, it is also worth noting that some idealizations suggested in the literature will not help. For instance, *Ten* is a judgement about Pythagoras’s whole life, which he holds robustly; and thus restrictions to ‘global’ (Griffin 1988: 105; see discussion in Heathwood 2014: 213, Raibley 2012) or ‘stable’ attitudes (Raibley 2010, Tiberius 2018, Tiberius and Plakias 2010) will not help. Additionally, assume Pythagoras gets pleasure from satisfying *Ten* in different domains, e.g. he feels pleased when he thinks about the fact that he has read fewer than ten books in his life. Thus, more complex accounts of what it means to value something which includes an affective component (e.g. Tiberius 2018, Tiberius and Plakias 2010) are also vulnerable.

## 3. Possible solutions to the new paradox

I now consider two possible idealizations that subjectivists might adopt to avoid the new paradox. These two idealizations are adopted by Dorsey, and it is his formulations that I engage. However, they are also two obvious routes for other subjectivists, and I consider deviations from Dorsey’s theory where this makes a difference. The two idealizations are that value judgements must be ‘coherent’ and ‘considered’.

Take coherence first*. Ten* is not internally incoherent. There is nothing self-contradictory about it; we can understand what Pythagoras means when he makes the judgement, and it is satisfiable in many cases. For a coherence requirement to help, we need a more complex understanding. Dorsey (2021: 144) provides one. According to his view, *S*’s well-being is grounded by what *S* ‘values’ (see also Raibley 2010, Tiberius 2018 and Tiberius and Plakias 2010 though these are each more complex than Dorsey’s account); and it is in determining what *S* values that we consult *S*’s prudential judgements. Coherence requires that ‘one’s evaluative judgements should not offer inconsistent evaluative verdicts concerning individual bearers of intrinsic prudential value’ (2021: 144). Thus, judgements should not just be internally coherent, but also *mutually* coherent.

Mutual coherence is not joint satisfiability – I can coherently judge it good that I get two different cakes from the baker but be unable to afford both – but rather coherence *in judgement*. A person’s coherent set of value judgements is determined through ‘minimal mutilation’, eliminating weaker or more peripheral judgements first (2021: 145). Dorsey imagines someone (call her the Gourmand) who judges it intrinsically prudentially good to eat food from Julia Child recipes and intrinsically prudentially bad to eat French cooking. Since Child’s recipes are French, these judgements are mutually, though not internally, incoherent. The Gourmand values and disvalues the same thing at the same time, under different descriptions. If we tell the Gourmand that Julia Child’s recipes are all French, she should adjust her judgements.

If coherence is to rescue JS from the new paradox, the tension involved should be more like the Gourmand tension than the bakery tension. In other words, it should be a genuine incoherence, not simply a case where mutually coherent judgements cannot be jointly satisfied.

To fail this more demanding coherence test, *Ten* must face tension with some other value judgement or with itself under another description. And indeed, the new paradox does seem to involve considering a particular event under different descriptions. If we ask Pythagoras whether he judges it prudentially good that *Ten* is satisfied, he will say yes. If we ask him whether he wants his *tenth* judgement to be satisfied, he will say no. We can present him with the fact that *Ten* is his tenth judgement, similarly to presenting the Gourmand with the fact that Child’s cooking is French.

But these tensions are importantly different. Remember that in some cases, such as wanting two cakes but only having money for one, we cannot get all the things we judge to be good for us due to circumstance. There is no inherent tension between having cakes A and B; but if I get cake A I cannot get cake B, and vice versa. If things were different – if the cakes were cheaper or I had more money – I could satisfy both judgements. For the Gourmand, there are no circumstantial changes that would enable them to satisfy both judgements.

In this respect, Pythagoras’s judgement is more like the bakery example than the Gourmand. There are circumstances under which Pythagoras can satisfy *Ten*. If he had only satisfied eight prudential judgements, he would be able to coherently satisfy *Ten*. It is bad luck that his circumstances mean he cannot satisfy it. And the reason he cannot satisfy it is because of its relation to his nine other satisfied judgements. *Ten* is satisfiable under the right conditions; but it is not *jointly* satisfiable with the nine other prudential judgements Pythagoras made previously.

Moreover, for the Gourmand, the solution is obvious: she must either change her mind about French food (‘Julia Child cooks *French* food? Turns out I do like it!’), or Child (‘Julia Child’s been cooking *French* food this whole time? Disgusting!’). But there is no straightforward resolution to the paradox of judgement. In the circumstances, there is no coherent way for Pythagoras to abandon just one of two evaluative attitudes that have come into tension, since the problem comes not from an incoherence among different value attitudes, but from the same event both satisfying and frustrating the *same* value attitude.

A defender of judgement subjectivism might object here that an important difference between Pythagoras’s situation and the bakery customer’s is that although the customer cannot jointly satisfy her judgements, they do still fit together. The problem with *Ten* is different: although not essentially incoherent, it turns out to be *self-defeating* in some contingent circumstances. Some subjectivists might insist that this contingent self-defeat is a kind of incoherence which also rules an attitude out of grounding well-being. One observation that potentially supports this is that while the case of Epimenides is not paradoxical for non-subjectivist views of well-being, the case of Pythagoras retains its paradoxical nature independently of its implications for well-being. For instance, a hedonist about well-being will still see that there is a puzzle about whether *Ten* is satisfied or not, even if they regard this puzzle as irrelevant to well-being.

It is worth thinking about what this means for Pythagoras’s attitudes. One option is that Pythagoras can abandon the judgement *Ten*. If he did this, there would be no tension. However, this is unsatisfying, at least if we accept Dorsey’s theory in full. Dorsey’s concern with coherence is not a *post hoc* attempt to tidy up our evaluative sets so they fit neatly into theory; rather, it is an attempt to get at what we really value. It seems reasonable to say that the Gourmand either did really value some French cooking, or did not really value Julia Child’s recipes; dropping a judgement is a way of getting at what she really values. But for Pythagoras, this is not true. If we say to him, ‘Look, your judgement is paradoxical. You must have made a mistake about what you value’, it would be reasonable for Pythagoras to insist there has been no mistake – he really does value avoiding going above ten – he has just been unlucky. Dropping the value judgement *Ten* might avoid the paradox; but it is not a good way of getting at what he really values.

Still, I have claimed that this new paradox is a problem for a wide variety of subjectivist views. Even if *Dorsey’s* theory does not sit well with stipulating that the judgement *Ten* (or some equivalent attitude) is to be excluded in problematic cases, a different subjectivist could make this stipulation. One such example is Tiberius’s (2018) value theory, which may endorse the stronger version of mutual coherence as joint satisfiability that Dorsey rejects. Tiberius says that our well-being is determined by how well we satisfy our ‘appropriate’ values, where appropriate values are, *inter alia*, ‘capable of being fulfilled together over time’. This is amenable to weaker and stronger readings. On a weaker reading, it is enough that two values are *in principle* mutually fulfillable, even if the real world makes doing so impossible. On this reading, Tiberius’s view of mutual coherence is close to Dorsey’s. But on a stronger reading, values cease to be appropriate if they cannot *in fact* be fulfilled together. For instance, if someone values becoming a professional chef but also values becoming an accountant then, to the extent that it is not possible to be both, at least one ceases to be an ‘appropriate’ value. Thus, it may be that *Ten* becomes an inappropriate value simply by virtue of being *de facto* unfulfillable.

I suspect this reading is too strong. Assume that values-based views also hold that *not* getting what we value is bad for us (this is not strictly required by a view that holds that getting what we value is good for us, but it is a natural extension of it). If we adopted the stronger reading of joint satisfiability, this would mean that whenever one value clashes with another this is not really bad for our well-being because whichever value is not fulfilled is ‘inappropriate’, and thus irrelevant to well-being. That would imply – implausibly, I think – that there are never well-being trade-offs in making difficult choices, such as a choice over what career to pursue. For instance, if you choose to become a chef while still also valuing becoming an accountant, the stronger reading implies that it is not at all intrinsically bad for you to have this latter value go unsatisfied. But precisely what makes such choices difficult is that they *do* require giving up things we value; even if this is net positive for our well-being, there is *some* cost to doing so.

Thus, although on this reading Tiberius’s theory may escape the paradox, I suggest that it does so at the cost of problems elsewhere. Note that this is different from the claim that the *best* life will be one where all one’s values are jointly satisfiable: at other points Tiberius (2018: 57, 68; see also Raibley 2012: 252) seems to have this question in mind. *Ten* is not conducive to a good life; it makes it harder for the other things Pythagoras values to promote his well-being. But this is a different question than whether the judgement *Ten*, given Pythagoras actually does make it, is admissible as a welfare-*grounding* judgement.

Finally, a subjectivist might exclude judgements simply when they *risk* the kind of paradoxical result I have outlined. On this view, even if Pythagoras had only fulfilled eight other value judgements, *Ten* could not contribute to his welfare even though it is non-paradoxically satisfiable. Again, I acknowledge this as a possibility. But it is important to have an external motivation for such exclusions beyond the avoidance of paradox, and I am unsure why the risk is enough to exclude *Ten* in cases where it does not result in paradox.

Thus, the situation Pythagoras finds himself in seems to me to be a temporally extended, and numerically expanded, version of the situation where I can only buy one of two cakes. Pythagoras’s situation is not like the tension involved in valuing Julia Child’s food but disvaluing French food.

Turn now to a second idealization. Dorsey also says that the judgements which ultimately ground facts about well-being must be ‘considered’. The reason for the consideration requirement relates to the role of what a person values in JS; Dorsey suggests that if *S* takes a valuing attitude towards something, that is not enough to say they really value that outcome. For instance, he imagines someone who claims to value becoming US President, but only because they misunderstand what being President would be like. Were they to learn what the Presidency really involves they would no longer value it. Thus, says Dorsey, they do not actually value becoming President even before learning the truth (see also Raibley 2010: 607–8). Rather, they value becoming President given the conditions they imagine it to involve. Dorsey suggests that *S* does not really value a particular thing ‘in and of itself’ if they ‘take the relevant valuing attitude toward [it] under a particular description, but don’t take that same valuing attitude under some other description’ (2021: 150).

What does this mean in practice? Dorsey considers Sobel’s (2009: 337) suggestion that the pro-attitudes relevant to well-being must be those we would make on the basis of full information, rejecting this as insufficient because it is too tethered to the actual world. Dorsey’s (2021: 150) ‘full consideration’ condition is that ‘a necessary condition for *x* to value 𝜙 is that *x would* take the relevant attitude toward 𝜙 given full consideration of the ways 𝜙 might be’. If I value becoming President in a wide range of conditions, but not under conditions in which I have children, Dorsey’s analysis suggests that I do not value becoming President, but rather value something else, such as ‘becoming President while childless’ (see also Tiberius and Plakias 2010: 422–23).

Let us return to Pythagoras. There are various reasons he might have for forming the judgement *Ten*. For instance, he might think the number ten is unlucky, and going above it will make bad things happen. Since that is untrue, full consideration would rule out Pythagoras’s attitude as grounding well-being. However, this explanation of Pythagoras’s aversion to the number ten is irrelevant to the paradox, since the relevant judgement concerns instrumental rather than intrinsic prudential badness.

Here is a more relevant story. Imagine that Pythagoras developed an instrumental ‘bad luck’ judgement such as the one mentioned just above early on in his life, and believed that exceeding the number ten would cause bad things to happen. However, the attitudes and habits he developed around this instrumental belief became so central to his life that he now holds an *intrinsic* prudential attitude towards the number ten. Ten is a special number, he now thinks, to be avoided for its own sake. He has forgotten the childish origins of his belief. And that new belief is now so deeply rooted that revealing the truth – that failing to avoid the number ten will not cause any harm – will not shake him from his judgement that it is to be avoided. Nor will reminding him of the origins of his judgements.

Such an attitude seems irrational. But it is not, I think, unconsidered in Dorsey’s sense. Indeed, the sense that it is irrational is most obviously explained as a judgement that exceeding the number ten is *not intrinsically bad* for anyone. However, Dorsey cannot appeal to this as one of the facts which full consideration would reveal to Pythagoras, since it is precisely this sort of claim that is decided by each individual’s values on JS.

I noted earlier that in the circumstances in which Pythagoras finds himself, there is a tension in satisfying the judgement *Ten*. I suggested that although there was such a tension, it was not of the sort needed for Dorsey’s coherence condition to rescue JS from the paradox of judgement. One might think that this tension is, however, susceptible to the consideration condition. The line of thought may run as follows: Pythagoras thinks he values ‘avoiding having ten or more of my prudential judgements satisfied’. But under conditions of full consideration, he would have to consider the following circumstance: ‘avoiding having ten or more of my prudential judgements satisfied *where doing so satisfies my tenth satisfied judgement*’. And, one might think, Pythagoras will reject satisfying his tenth judgement where this frustrates that very judgement.

However, I think such a response misunderstands the tension at the heart of the paradox. The problem raised by the paradox is unlike Dorsey’s case of becoming President, where things are not the way the valuing agent expects. If Pythagoras is thinking straight, when we ask him whether he values satisfying *Ten* in circumstances where he has already satisfied nine such judgements, he should not reply ‘yes’ or ‘no’. He should rather reply that it is simply not clear whether it is possible to satisfy *Ten* in this case. The basic problem with the paradox of judgement is not that the ‘less-than-ten’ judgement is satisfied in circumstances that make it unattractive, but that there is a paradox in the very question of whether it is satisfied. And it is *this* paradox that leads, for JS, to a paradox in determining Pythagoras’s overall lifetime well-being.

I suggest, then, that it is possible for Pythagoras to value avoiding the number ten as far as possible even following full consideration, and that appeals to full consideration will not help resolve the tension at the heart of the paradox of judgement.

I have argued that a wide range of subjectivist theories are vulnerable to paradoxical implications in unusual but conceivable circumstances, including views based on prudential value judgements which avoid the paradox of desire. If it is a requirement of a theory of well-being that it has no paradoxical implications, then many subjectivist theories fail. Subjectivists must either add an additional idealization that can solve the new paradox or explain why such paradoxes do not constitute serious objections to a theory of well-being.^4^

## Footnotes

1 The suggestion is not that the same fact could not be both intrinsically good for you and intrinsically bad for you. The incoherence is in the thought that it is *the fact that it is intrinsically bad for you* which is intrinsically good for you.

2 This paradox stems from a discussion with Clayton Littlejohn (drawing on work by Bacon (e.g. 2021)) about a related paradox for the accuracy of seemings.

3 Readers may worry such ascriptions are not intrinsic. I address this below.

4 Thanks to Guy Fletcher, Alex Gregory and two anonymous referees for helpful feedback.

## Notes

Thanks to Guy Fletcher, Alex Gregory and two anonymous referees for helpful feedback.

## References

[CIT0001] Bacon, A. 2021. Opacity and paradox. In Modes of Truth: The Unified Approach to Truth, Modality, and Paradox, eds. C. Nicolai and J. Stern, 231–65. Abingdon: Routledge.

[CIT0002] Barrett, J. 2019. Interpersonal comparisons with preferences and desires. Politics, Philosophy & Economics 18: 219–41.

[CIT0003] Barrett, J. 2022. Subjectivism and degrees of well-being. Utilitas 34: 97–104.

[CIT0004] Bradley, B. 2007. A paradox for some theories of welfare. Philosophical Studies 133: 45–53.

[CIT0005] Bradley, B. 2009. Well-being and Death. Oxford: Oxford University Press.

[CIT0006] Dorsey, D. 2012. Subjectivism without desire. Philosophical Review 121: 407–42.

[CIT0007] Dorsey, D. 2021. A Theory of Prudence. Oxford: Oxford University Press.

[CIT0008] Feldman, F. 2004. Pleasure and the Good Life: Concerning the Nature, Varieties, and Plausibility of Hedonism. Oxford: Clarendon Press.

[CIT0009] Griffin, J. 1988. Well-Being: Its Meaning, Measurement, and Moral Importance. Oxford: Oxford University Press.

[CIT0010] Heathwood, C. 2005. The problem of defective desires. Australasian Journal of Philosophy 83: 487–504.

[CIT0011] Heathwood, C. 2006. Desire satisfactionism and hedonism. Philosophical Studies 128: 539–63.

[CIT0012] Heathwood, C. 2014. Subjective theories of well-being. In The Cambridge Companion to Utilitarianism, eds. B. Eggleston and D. Miller, 199–219. Cambridge: Cambridge University Press.

[CIT0013] Heathwood, C. 2022. Happiness and desire satisfaction. Noûs 56: 57–83.

[CIT0014] Lin, E. 2016. The subjective list theory of well-being. Australasian Journal of Philosophy 94: 99–114.

[CIT0015] Murphy, M. 1999. The simple desire‐fulfillment theory. Noûs 33: 247–72.

[CIT0016] Raibley, J.R. 2010. Well-being and the priority of values. Social Theory and Practice 36: 593–620.

[CIT0017] Raibley, J.R. 2012. Welfare over time and the case for holism. Philosophical Papers 41: 239–65.

[CIT0018] Sobel, D. 2009. Subjectivism and idealization. Ethics 119: 336–52.

[CIT0019] Tiberius, V. 2018. Well-Being as Value Fulfilment: How We Can Help Each Other to Live Well. Oxford: Oxford University Press

[CIT0020] Tiberius, V. and A. Plakias. 2010. Well-being. In The Moral Psychology Handbook, ed. J. Doris, 402–32. Oxford: Oxford University Press.

